# Supplementary material for: A Critical View of the Application of the APEX Software (Aqueous Photochemistry of Environmentally-Occurring Xenobiotics) to Predict Photoreaction Kinetics in Surface Freshwaters
Source: Molecules. 2019 Dec 18;25(1):9. doi: 10.3390/molecules25010009 (PMC7017383; doi:10.3390/molecules25010009)
Supplement: Supplementary file 1 [file molecules-25-00009-s001.zip › 3.2.4_gcc-4.4.0.zip!/3.2.4_gcc-4.4.0/3.2.4_gcc-4.4.0/doc/octave/html/interpreter/Linear-Least-Squares.html]

Linear Least Squares - Untitled


Previous: Nonlinear Programming,
Up: Optimization


---

### 24.4 Linear Least Squares

Octave also supports linear least squares minimization. That is,
Octave can find the parameter b such that the model
y = x\*b
fits data (x,y) as well as possible, assuming zero-mean
Gaussian noise. If the noise is assumed to be isotropic the problem
can be solved using the \ or / operators, or the `ols`
function. In the general case where the noise is assumed to be anisotropic
the `gls` is needed.

— Function File: [beta, sigma, r] = **ols** (y, x)  
> Ordinary least squares estimation for the multivariate model
> y = x b + e with
> mean (e) = 0 and cov (vec (e)) = kron (s, I).
> where
> y is a t by p matrix, x is a t by
> k matrix, b is a k by p matrix, and
> e is a t by p matrix.
>
> Each row of y and x is an observation and each column a
> variable.
>
> The return values beta, sigma, and r are defined as
> follows.
>
> beta: The OLS estimator for b, beta `= pinv (`x`) *` y, where `pinv (`x`)` denotes the pseudoinverse of x. sigma: The OLS estimator for the matrix s, ``` sigma = (y-x*beta)' * (y-x*beta) / (t-rank(x)) ``` r: The matrix of OLS residuals, r `=` y `-` x `*` beta.

— Function File: [beta, v, r] = **gls** (y, x, o)  
> Generalized least squares estimation for the multivariate model
> y = x b + e with mean (e) = 0 and
> cov (vec (e)) = (s^2) o,
> where
> y is a t by p matrix, x is a t by
> k matrix, b is a k by p matrix, e
> is a t by p matrix, and o is a t p by
> t p matrix.
>
> Each row of y and x is an observation and each column a
> variable. The return values beta, v, and r are
> defined as follows.
>
> beta: The GLS estimator for b. v: The GLS estimator for s^2. r: The matrix of GLS residuals, r = y - x beta.

— Function File: x = **lsqnonneg** (c, d)  
— Function File: x = **lsqnonneg** (c, d, x0)  
— Function File: [x, resnorm] = **lsqnonneg** (...)  
— Function File: [x, resnorm, residual] = **lsqnonneg** (...)  
— Function File: [x, resnorm, residual, exitflag] = **lsqnonneg** (...)  
— Function File: [x, resnorm, residual, exitflag, output] = **lsqnonneg** (...)  
— Function File: [x, resnorm, residual, exitflag, output, lambda] = **lsqnonneg** (...)  
> Minimize `norm (`c`*`x`-d)` subject to x `>=
> 0`. c and d must be real. x0 is an optional
> initial guess for x.
>
> Outputs:
>
> - resnorm
>
>   The squared 2-norm of the residual: norm(c\*x-d)^2- residual
>
>     The residual: d-c\*x- exitflag
>
>       An indicator of convergence. 0 indicates that the iteration count
>       was exceeded, and therefore convergence was not reached; >0 indicates
>       that the algorithm converged. (The algorithm is stable and will
>       converge given enough iterations.)- output
>
>         A structure with two fields:
>         - "algorithm": The algorithm used ("nnls")- "iterations": The number of iterations taken.
>         - lambda
>
>           Not implemented.
>
>
>
>
> **See also:** optimset.

— Function File: **optimset** ()  
— Function File: **optimset** (par, val, ...)  
— Function File: **optimset** (old, par, val, ...)  
— Function File: **optimset** (old, new)  
> Create options struct for optimization functions.

— Function File: **optimget** (options, parname)  
— Function File: **optimget** (options, parname, default)  
> Return a specific option from a structure created by
> `optimset`. If parname is not a field of the options
> structure, return default if supplied, otherwise return an
> empty matrix.
